# Pyruvate carboxylase promotes malignant transformation of papillary thyroid carcinoma and reduces iodine uptake

**DOI:** 10.1038/s41420-022-01214-y

**Published:** 2022-10-20

**Authors:** Yang Liu, Chang Liu, Yu Pan, Jinxin Zhou, Huijun Ju, Yifan Zhang

**Affiliations:** Department of Nuclear Medicine, Ruijin Hospital, Shanghai Jiaotong University, School of Medicine, Shanghai, 200025 China

**Keywords:** Tumour biomarkers, Head and neck cancer, Metastasis

## Abstract

Previous studies have shown that pyruvate carboxylase (PC) plays a key role in the occurrence and progression of thyroid cancer (TC); however, the relationship between PC and iodine-refractory TC is unclear. Therefore, the present study aimed to investigate the molecular mechanism of PC in the malignant progression and loss of iodine uptake in papillary TC (PTC) and to explore the potential therapeutic effect of PC inhibitors in iodine-refractory PTC. PC increased cell proliferation, invasion, and metastasis, inhibited expression of the iodine metabolism-related genes *TSHR*, *NIS*, *TPO*, and *TG*, and decreased the iodine-uptake capacity by activating the mitogen-activated protein kinase/extracellular signal-regulated kinase pathway in PTC cell lines. Furthermore, the PC inhibitor ZY-444 effectively inhibited the activation of PC, reduced the malignant invasiveness, and restored the expression of iodine metabolism-related genes and the iodine-uptake capacity in PTC cells. These findings suggest that PC activation is involved in the progression of iodine-refractory TC and that PC inhibitors may represent a potentially novel targeted therapy for iodine-refractory TC.

## Introduction

Thyroid cancer (TC) is the most common malignant tumor of the endocrine system, and its incidence has recently been increasing [1]. Papillary TC (PTC) is the most frequent type of TC [2] and has a relatively good prognosis after treatment, including surgery, radioactive iodine-131, and thyroid-stimulating hormone suppression therapy [3]. However, 20–30% of patients with metastases still develop resistance to radioactive iodine-131 treatment, referred to as dedifferentiation, which manifests as reduced or no iodine uptake in the lesions, increased tumor invasiveness and metastasis, and eventual progression to radioiodine-refractory differentiated TC (RR-DTC) [4], with a 10-year survival rate of only 10% [5, 6].

Metabolic reprogramming is known to be an important characteristic of tumor occurrence and progression [7]. Pyruvate carboxylase (PC) is a biotin-dependent mitochondrial enzyme that plays an important role in metabolic processes in eukaryotes [8]. Strickaert et al. reported that PC was a key enzyme affecting the proliferation, invasion, and metastasis capacity of PTC, and was involved in supplementing the tricarboxylic acid cycle [9]. In addition, we previously showed that PC expression was higher in PTC tissues and thyroid fine-needle aspiration washout from patients with central lymph node metastasis than in patients without central lymph node metastasis [10, 11]. However, the relationship between PC and iodine uptake in TC remains unclear.

In addition, TC is primarily a mitogen-activated protein kinase (MAPK) pathway-driven cancer. The MAPK signaling pathway (RET–RAS–RAF–MEK–ERK) regulates tumor growth, invasion, metastasis, and differentiation by regulating the phosphorylation of multiple substrates during the development of TC [12], and is considered as the main pathway responsible for malignant progression and loss of iodine accumulation in TC [13, 14]. TC dedifferentiation involves abnormal silencing of iodine metabolism-related genes, such as thyroid-stimulating hormone receptor (TSHR), Na^+^/I^−^symporter (NIS), thyroid peroxidase (TPO), and thyroglobulin (TG), which are associated with MAPK pathway activity [15, 16]. Kinase inhibitors targeting the major protein components of this signaling cascade have recently been developed and used as targeted drugs to treat RR-DTC, with good efficacy. However, drug resistance and adverse reactions are prone to occur during the treatment process, and further investigations are therefore needed to determine the molecular mechanisms responsible for the dedifferentiation process and to identify new drug targets. The small-molecule metabolic compound ZY-444 has recently been shown to bind to PC and inhibit its activity, and to play a role in inhibiting tumor proliferation and metastasis in breast cancer [17]. However, the clinical significance and functional roles of ZY-444 in TC remain to be elucidated.

This study aimed to explore the molecular mechanism underlying the role of PC in the malignant progression of PTC and loss of iodine uptake by assessing its effects on the proliferation, metastasis, and iodine uptake of PTC cells. We also assessed the abilities of a novel PC inhibitor to inhibit tumor invasion and metastasis and improve tumor iodine uptake, thus allowing further iodine-131 therapy. The results of this study will further our understanding of PTC.

## Results

### Regulation of PC expression in PTC cell lines

**Fig. 1:**
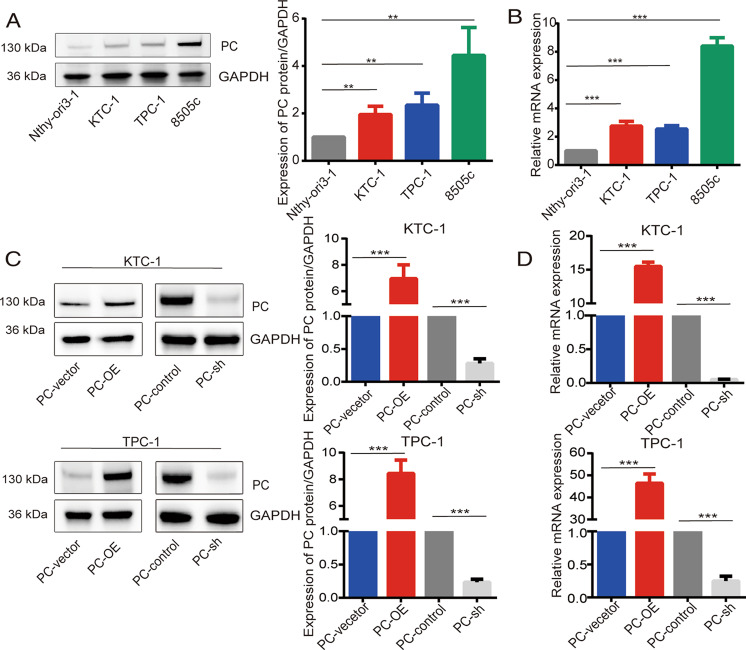
PC expression in TC and normal cells and in TPC-1 and KTC-1 cells stably transfected with PC lentivirus. **A** PC protein detected by western blot and **B** PC mRNA expression detected by RT-PCR in normal thyroid epithelial cells and TC cell lines. **C** PC protein detected by western blot and **D** PC mRNA expression detected by RT-PCR in TPC-1 and KTC-1 cells stably transfected with PC lentivirus. *PC* gene was knocked down by lentivirus-PCshRNA (PC-sh) and overexpressed by lentivirus-PC (PC-OE). *p < 0.05, **p < 0.01, ***p < 0.001. Quantitative results based on three independent experiments. Protein expression quantified by ImageJ analysis of western blots. Full blots provided in the Supplementary File.

We first assessed the expression of PC in normal thyroid epithelial cells and different TC cell lines. Quantitative real-time (RT)-PCR and western blot were used to detect PC mRNA and protein expression levels, respectively, in two PTC cell lines (TPC-1 and KTC-1), the anaplastic TC cell line (8505c), and an immortalized normal thyroid epithelial cell line (Nthy-ori3-1). PC expression levels were highest in 8505c while lower in TPC-1 and KTC-1, and lowest in Nthy-ori3-1, in terms of both protein expression (*p* < 0.01) and mRNA expression (*p* < 0.001; Fig. 1A, B). We therefore selected the PTC cell lines TPC-1 and KTC-1 and stably transfected them with lentiviruses to induce PC overexpression or knockdown for subsequent experiments. Compared with vector mock-transfected control cells, PC protein and mRNA expression levels were significantly increased in PC-overexpressing TPC-1-PC-OE and KTC-1-PC-OE cells and decreased in PC-knockdown TPC-1-sh and KTC-1-sh cells (all *p* < 0.001; Fig. 1C, D).

### PC promotes the proliferation, invasion, and migration of PTC cells

**Fig. 2:**
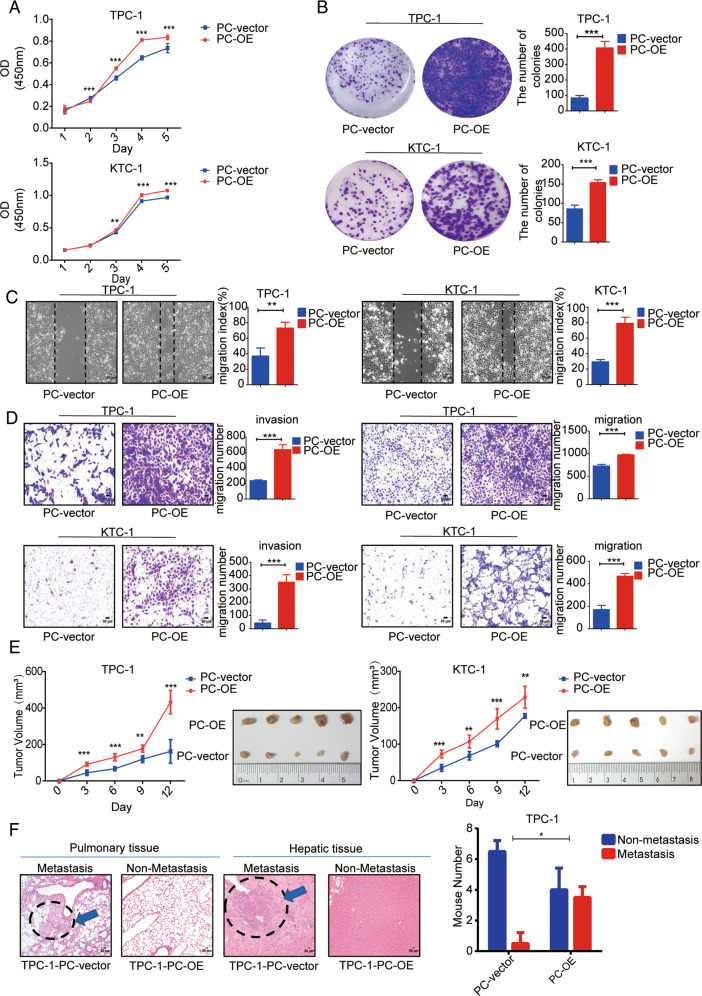
PC promotes aggressiveness of PTC cells. **A** Cell proliferation abilities of TPC-1 and KTC-1 cells detected by CCK8 assay. **B** Colony-formation capacities of TPC-1 and KTC-1 cells assessed by clonogenic assay. Metastatic abilities of TPC-1 and KTC-1 cells detected by **C** scratch assay and **D** Transwell migration and invasion assays, respectively. **E** TPC-1 and KTC-cells tumor xenograft volumes in nude mice. Right: representative image of explanted tumors. Each group comprised five mice. **F** Hematoxylin-eosin-stained tumors showing lung and live metastases after tail injection of TPC-1-vector or TPC-1-PC-OE cells. Two mice in the TPC-1-PC-OE developed liver metastases as well as lung metastases. Right: graph showing numbers of mice with lung metastases. Each group comprised eight mice. The experiment was repeated twice. ***p < 0.001, **p < 0.01, *p < 0.05. Representative photomicrographs taken at ×100 magnification. Results expressed as mean ± SD of three independent experiments and shown in histograms on the right. **F** shows results of *χ*^2^ test.

We determined the effect of PC on the biological behavior of PTC cells. The proliferation abilities of the two PTC cell lines were significantly higher than that of the control cells on the third day after PC overexpression, as shown by CCK-8 assay (*p* < 0.001; Fig. 2A). In addition, PC overexpression promoted colony formation in TPC-1 and KTC-1 cells, as shown by clonogenic assay (*p* < 0.001; Fig. 2B). Scratch wound assay and transwell assays showed that the migration abilities of the two PC -overexpressed PTC cell lines were significantly enhanced compared with the control cells (*p* < 0.001; Fig. 2C, D). In addition, PC knockdown inhibited cell proliferation, colony formation, and migration of PTC cell lines (Fig. S1).

The proliferation rate and tumor size of TPC-1 and KTC-1 tumor xenografts in nude mice in vivo were significantly higher within 12 days after overexpression of PC compared with control xenografts (Fig. 2E). We detected the metastatic ability of tumors in vivo by tail vein injection. Further dissection and hematoxylin and eosin staining of the liver and lungs revealed that significantly more mice with the PC-overexpressing TPC-1 group developed lung metastases compared with the control group(*p* < 0.05), while two mice in the PC-overexpressing TPC-1 group also developed liver metastases along with lung metastases (Fig. 2F). These results suggested that PC could enhance cell proliferation, invasion, and migration in vivo and in vitro, thereby promoting tumor occurrence and progression.

### PC reduces iodine metabolism-related gene expression and iodine uptake and activates the MAPK pathway

**Fig. 3:**
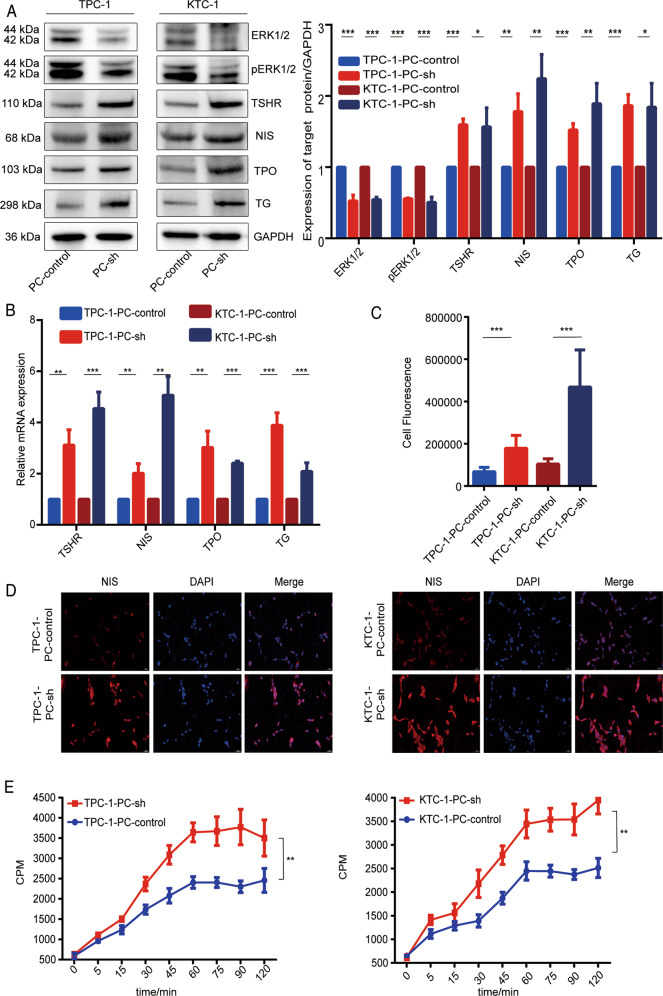
PC reduces expression of iodine metabolism-related genes and iodine uptake and promotes MAPK pathway signaling in PTC cells. **A** Protein and **B** mRNA expression levels of TSHR, NIS, TPO, TG, and MAPK pathway signals in TPC-1 and KTC-1 cells, detected by western blot and RT-PCR, respectively. Protein expression quantified by ImageJ analysis of western blots. Full blots provided in the Supplementary File. **C** Quantification of NIS fluorescence. **D** Immunofluorescence detection of localization and expression of NIS in TPC-1 and KTC-1 cells (magnification ×20 objective); **E** Iodine uptake detected by ^125^I uptake assay in TPC-1 and KTC-1 cells. ***p < 0.001, **p < 0.01, *p < 0.05. Quantitative results based on three independent experiments.

We explored the effect of PC on iodine metabolism by examining its effect on the expression of genes related to iodine metabolism. RT-PCR and western blot analysis of TPC-1 and KTC-1 cell lines showed that mRNA and protein expression levels of TSHR, NIS, TPO, and TG were all significantly higher after PC knockdown (Fig. 3A, B) and decreased after PC overexpression (Fig. S2A–C). In addition, the fluorescence intensity of NIS was significantly higher in TPC-1-PC-sh and KTC-1-PC-sh cells compared with control cells (*p* < 0.001), and it was localized mostly on the cell membrane (Fig. 3C, D). PC knockdown significantly increased iodine uptake in both TPC-1 and KTC-1 cells (*p* < 0.01), as shown by the dynamic iodine-uptake curve (Fig. 3E).

PC overexpression significantly decreased the expression and function of iodine metabolism-related genes (Fig. S2A–D). Quantitative immunohistochemical analysis of tumor xenografts from nude mice showed that TSHR, NIS, TPO, and TG expression were all significantly reduced after overexpression of PC (Fig. S2E). In addition, iodine uptake by TPC-1 and KTC-1 in vitro and in vivo was also inhibited by PC overexpression (*p* < 0.01; Fig. S2F, G). These results suggest that PC can reduce iodine metabolism-related gene expression and iodine uptake in PTC cells.

To elucidate the possible mechanism by which PC reduces iodine uptake and promotes malignant transformation, we also investigated its effect on the expression of MAPK signaling pathway-related factors. Overexpression of PC significantly increased expression levels of the MAPK pathway-related factors mitogen-activated protein kinase kinase (MEK) 1/2, p-MEK1/2, extracellular signal-regulated kinase (ERK)1/2, and p-ERK1/2 in TPC-1 and KTC-1 cell lines compared with controls (Fig. S2B), while knockdown of PC decreased the expression of ERK1/2 and pERK1/2 (Fig. 3A). These results indicated that PC activated the MAPK pathway in TPC-1 and KTC-1 of PTC cell lines.

### Inhibition of ERK1/2 signaling restores iodine metabolism-related gene expression and iodine uptake

**Fig. 4:**
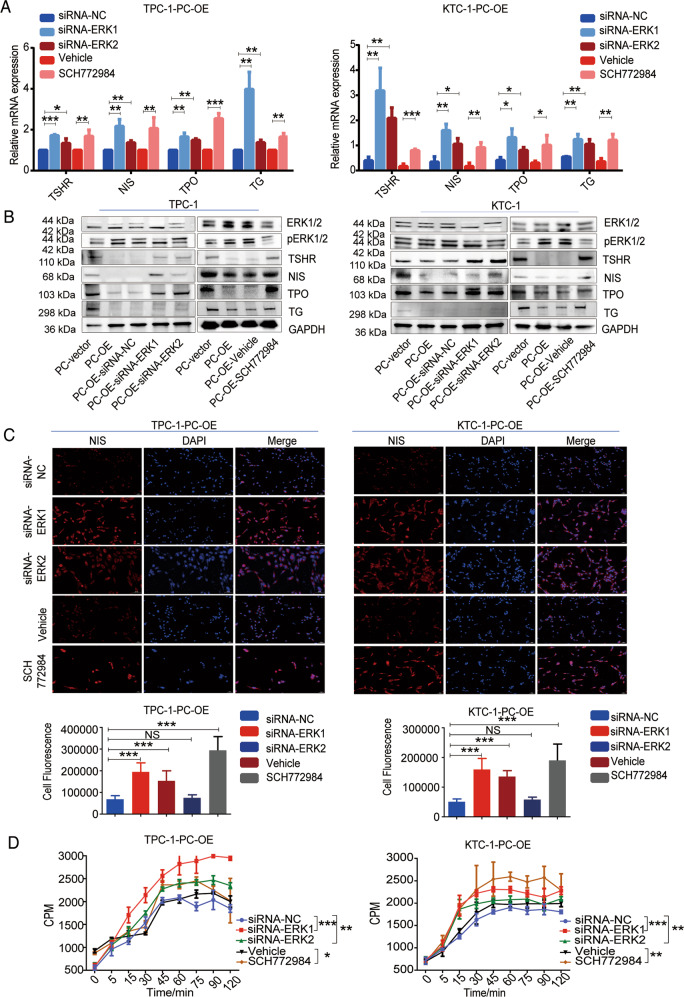
Reduced MAPK pathway signaling restores expression of iodine metabolism-related genes and iodine uptake in PC-overexpressed PTC cells. **A** mRNA expression of TSHR, NIS, TPO, and TG detected by RT-PCR and **B** protein expression of TSHR, NIS, TPO, TG, ERK1/2, and pERK1/2 detected by western blot in PC-overexpressed TPC-1 and KTC-1 cells with siRNAs or 5 µM SCH772984 knockdown of ERK1/2 signaling. Quantitative analysis of protein expression provided in Figure S6. Full blots provided in the Supplementary File. **C** Immunofluorescence detection and localization of NIS in PC-overexpressed TPC-1 and KTC-1 cells with siRNAs or 5 µM SCH772984 knockdown of ERK1/2 signaling (magnification 20× objective). Quantification of NIS fluorescence shown below. **D** Iodine uptake detected by ^125^I uptake assay in PC-overexpressed TPC-1 and KTC-1 cells with siRNAs or 5 µM SCH772984 knockdown of ERK1/2 signaling. ***p < 0.001, **p < 0.01, *p < 0.05. Results based on three independent experiments. Quantitative results represent three independent experiments.

To confirm the effect of PC on iodine uptake and malignant transformation of PTC via the MAPK/ERK signaling pathway, we downregulated ERK1/2 and p-ERK1/2 protein levels using siRNA-ERK1 and siRNA-ERK2 or the ERK inhibitor SCH772984. We detected TSHR, NIS, TPO, and TG mRNA expression levels by RT-PCR after treatment with SCH772984 or transfection of TPC-1-PC-OE and KTC-1-PC-OE cells with siRNAs. Inhibition of the MAPK/ERK signaling pathway restored the expression of these iodine metabolism-related genes in TPC-1-PC-OE and KTC-1-PC-OE cells (Fig. 4A). Western blot analysis of TSHR, NIS, TPO, and TG protein levels was consistent with their mRNA expression levels (Fig. 4B). These results suggest that downregulation of ERK1/2 signaling by siRNAs transfection or the use of a small-molecule inhibitor restored the expression of iodine metabolism-related genes in PC-overexpressing PTC cells. These results confirmed that PC inhibited the expression of PTC iodine metabolism-related genes by promoting activation of the MAPK pathway.

Immunofluorescence studies showed that transfection with siRNAs or treatment with the inhibitor SCH772984 inhibited ERK1/2 signaling in TPC-1-PC-OE and KTC-1-PC-OE cells, and increased intracellular NIS fluorescence intensity compared with the control group (*p* < 0.001) (Fig. 4C). The iodine-uptake capacity of TPC-1-PC-OE and KTC-1-PC-OE cells recovered significantly after treatment with the above siRNAs or ERK1/2 inhibitor (Fig. 4D).

### Inhibition of ERK1/2 signaling decreases cell proliferation, invasion, and migration

**Fig. 5:**
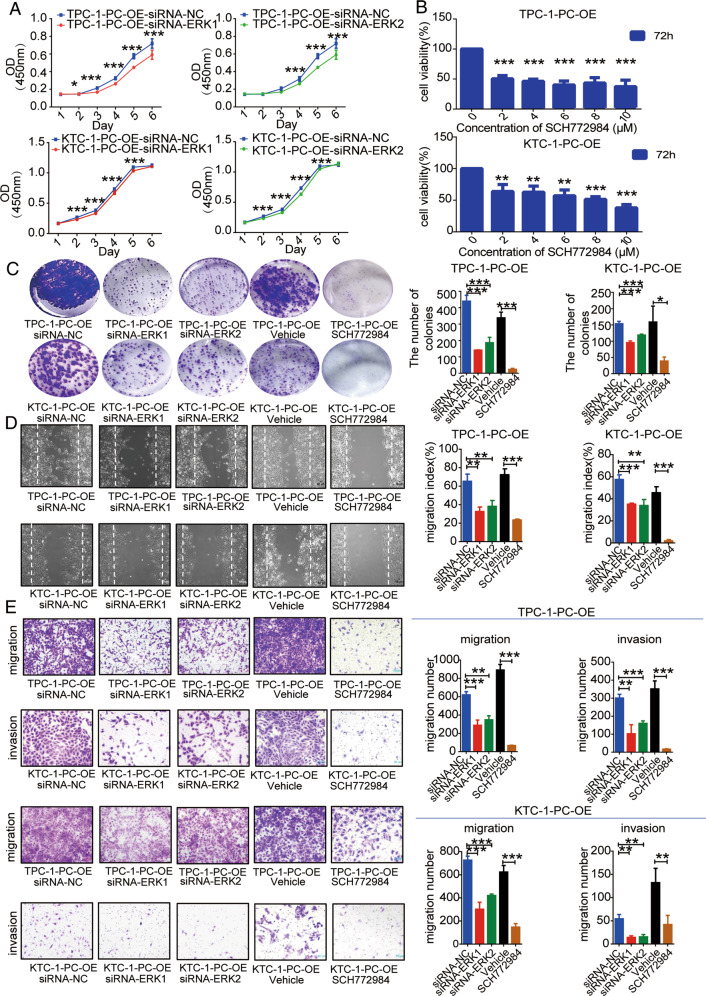
Reduced MAPK pathway signaling decreases aggressiveness of PC-overexpressed PTC cells. **A** Cell proliferation abilities of PC-overexpressed TPC-1 and KTC-1 cells with siRNAs knockdown of ERK1/2 signaling and **B** cell proliferation abilities of PC-overexpressed TPC-1 and KTC-1 cells with SCH772984 treatment detected by CCK8 assay. **C** Colony-formation ability of PC-overexpressed TPC-1 and KTC-1 cells with siRNAs or SCH772984 knockdown of ERK1/2 signaling. Metastatic abilities of PC-overexpressed TPC-1 and KTC-1 cells with siRNAs or SCH772984 knockdown of ERK1/2 signaling detected by **D** scratch assay and **E** Transwell migration and invasion assays, respectively. ***p < 0.001, **p < 0.01, *p < 0.05. Representative photomicrographs taken at 100× magnification. Results expressed as mean ± SD of three independent experiments and shown in histograms on the right.

The cell proliferation rate was significantly decreased from the 3rd or 4th day after transfection of TPC-1-PC-OE cells with siRNA-ERK1 or siRNA-ERK2, as shown by CCK-8 assay (*p* < 0.001). The proliferation rates of KTC-1-PC-OE cells were significantly decreased on the 2nd day after transfection with siRNA-ERK1 or siRNA-ERK2 (*p* < 0.001; Fig. 5A). The proliferation rates of TPC-1-PC-OE and KTC-1-PC-OE cells were also significantly decreased 72 h after treatment with the ERK inhibitor SCH772984 (Fig. 5B). Knockdown of ERK1/2 signaling by siRNAs or SCH772984 treatment further inhibited the colony-forming ability of TPC-1-PC-OE and KTC-1-PC-OE cells, as shown by clonogenic assay (*p* < 0.001; Fig. 5C). Scratch wound assay showed that downregulation of ERK1/2 signaling by siRNAs or SCH772984 also significantly inhibited the cell-migration capacity of TPC-1-PC-OE and KTC-1-PC-OE cells (Fig. 5D). Transwell assays also showed that the numbers of invasive and migrating cells were significantly reduced after transfection of TPC-1-PC-OE and KTC-1-PC-OE cells with siRNAs or treatment with the ERK inhibitor SCH772984 (Fig. 5E). Inhibition of ERK1/2 expression thus significantly inhibited cell proliferation, invasion, and migration in both TPC-1-PC-OE and KTC-1-PC-OE cells.

### Cell proliferation, invasion, and migration were inhibited by the PC inhibitor ZY-444

**Fig. 6:**
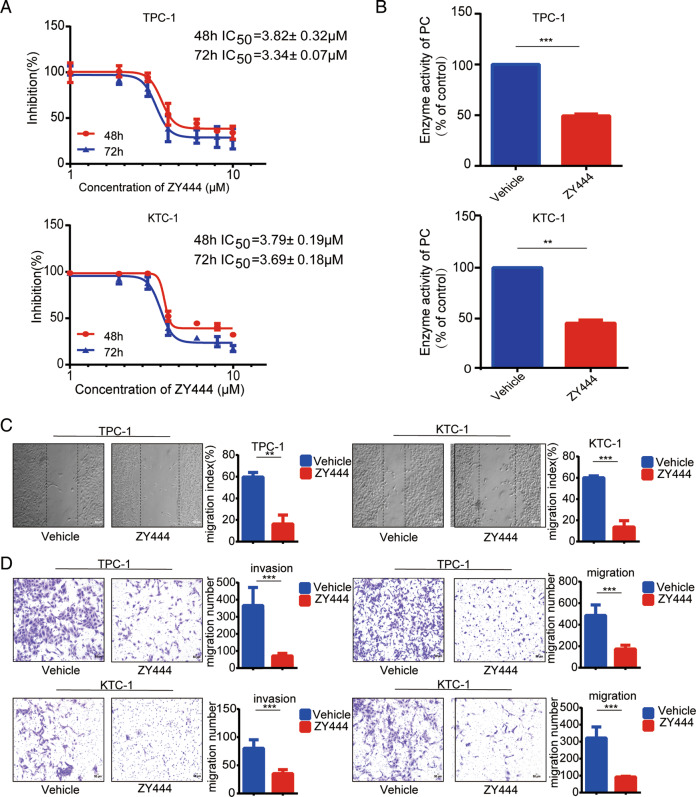
ZY-444 inhibits aggressiveness of PTC cells. **A** IC50 of TPC-1 and KTC-1 cells analyzed by CCK8 assay. **B** Effect of ZY-444 1 µM on PC enzymatic activity. Metastatic abilities of PC-overexpressed TPC-1 and KTC-1 cells with 1 µM ZY-444 treatment detected by **C** scratch assay and **D** Transwell migration and invasion assays, respectively. ***p < 0.001, **p <*p* < 0.01, *p < 0.05. Representative photomicrographs taken at ×100 magnification. Results expressed as mean ± SD of three independent experiments and shown in histograms on the right.

We treated TPC-1 and KTC-1 cells with the PC inhibitor ZY-444 to explore the potential of PC as a therapeutic target. Treatment with 4 μM ZY-444 for 48 h halved the proliferation abilities of TPC-1 and KTC-1 cells compared with the control group, as shown by CCK-8 assay (Fig. S3). The half-maximal inhibitory concentration (IC50) was calculated by curve fitting (Fig. 6A). We confirmed the effect of ZY-444 on PC activity and showed that ZY-444 significantly reduced PC activity in PTC cells at a concentration of 1 µM (Fig. 6B). Scratch wound assay and transwell results showed that ZY-444 significantly inhibited the invasion and migration capacity of TPC-1 and KTC-1 cells when the experimental concentration of ZY-444 used was 1 μM (Fig. 6C, D).

### ZY-444 restored iodine metabolism-related gene expression and iodine uptake in cells, by inhibiting the MAPK/ERK pathway

**Fig. 7:**
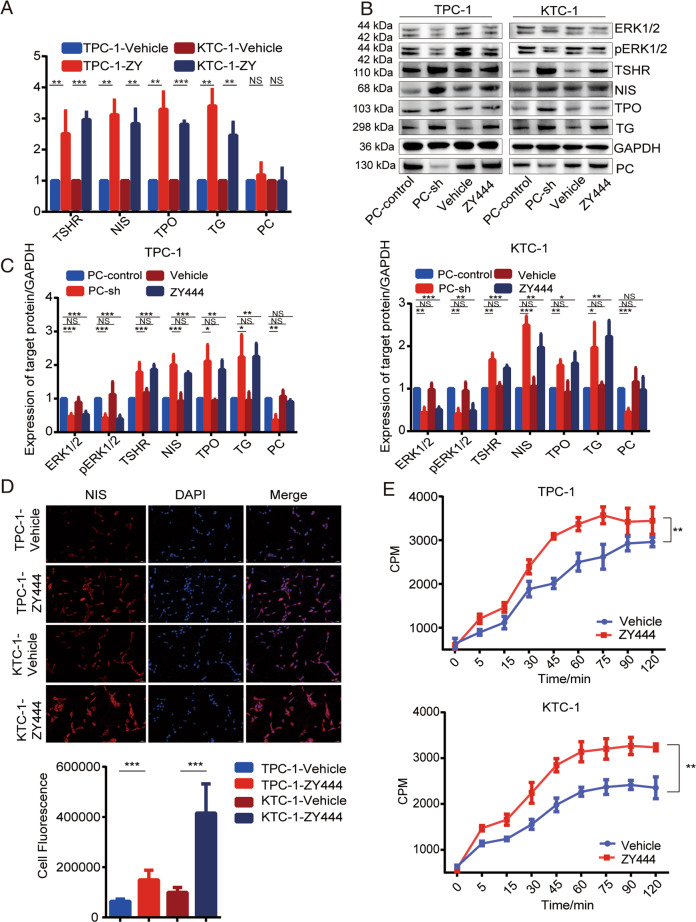
ZY-444 restores expression of iodine metabolism-related genes and iodine uptake in PTC cells. **A** mRNA expression of TSHR, NIS, TPO, and TG detected by RT-PCR and **B** protein expression of TSHR, NIS, TPO, TG, ERK1/2, and P-ERK1/2 detected by western blot in TPC-1 and KTC-1 cells with ZY-444 treatment. Full blots provided in the Supplementary File. **C** Quantitative analysis of protein expression. **D** Immunofluorescence detection and localization of NIS in TPC-1 and KTC-1 cells with ZY-444 treatment (magnification ×20 objective). Quantification of NIS fluorescence shown below. **E** Iodine uptake detected by ^125^I uptake assay in TPC-1 and KTC-1 cells with ZY-444 treatment. ***p < 0.001, **p < 0.01, *p < 0.05. Results expressed as mean ± SD of three independent experiments and shown in histograms on the right.

We investigated the effects of ZY-444 on expression levels of iodine metabolism-related genes and iodine uptake to determine if it could inhibit PC to alleviate iodine uptake and provide suitable conditions for iodine treatment. ZY-444 treatment of TPC-1 and KTC-1 cells significantly increased TSHR, NIS, TPO, and TG mRNA expression levels in TPC-1 and KTC-1 cells as shown by RT-PCR (Fig. 7A), and protein expression levels as shown by western blot (Fig. 7B, C). The fluorescence intensity of NIS on the cell membrane was also significantly higher in TPC-1 and KTC-1 cells treated with ZY-444 (*p* < 0.001; Fig. 7D), and ZY-444 significantly increased iodine uptake in TPC-1 and KTC-1 cells, as shown by iodine-uptake assay (Fig. 7E).

To verify the role of PC in PTC cells, we also showed that the expression and function of iodine metabolism-related genes were restored and cell-invasive behaviors were inhibited after inhibition of PC by ZY-444 in PC-overexpressing PTC cells (Figs. S4 and S5).

## Discussion

The incidence and prevalence of TC have been increasing in recent years. Although the prognosis of most patients is good, 1–4% of patients already have distant metastases at the time of their first diagnosis, and 7–23% of patients have distant metastases at a follow-up visit [18, 19]. One-third of patients with metastatic DTC show no radioactive iodine uptake at the time of their initial diagnosis, and two-thirds of patients with metastatic DTC convert to RR-DTC during the treatment process [14, 20]. There is often a lack of precise diagnostic targets and effective treatment methods for these patients.

PC is a mitochondrial enzyme that plays a key role in the process of pyruvate carboxylation. PC controls cancer growth via various methods, including modulation of glucose, lipid, and glutamine metabolism and modification of oxidative stress protection [21]. PC expression is enhanced in invasive breast cancer and plays an important role in tumor metabolism [22–25]. Furthermore, high expression of PC in breast cancer has been shown to affect tumor proliferation and metastasis, while PC expression was increased in lung metastases of breast cancer compared with the primary site, and knockdown of PC decreased NADPH/NADP^+^ ratios compared with untreated control cells and increased the sensitivity of breast cancer cells to H_2_O_2_ treatment, indicating that PC protected against reactive oxygen species (ROS) in tumor cells [26]. Strickaert et al. [9] demonstrated that tricarboxylic acid supplementation and tumor metabolic reprogramming both depended on enhanced PC expression in PTC. However, information on the relationship between PC and iodine uptake in PTC is lacking. Based on our previous studies [10, 11], we focused on the effect of PC on metastasis and iodine uptake in PTC, and aimed to provide valuable information regarding progression of the TC dedifferentiation process and RR-DTC.

The MAPK signaling pathway regulates tumor growth, invasion, metastasis, and differentiation by regulating the phosphorylation of multiple substrates during the development of PTC [27]. PTC iodine uptake is mediated by NIS, and the expression of radioactive iodine metabolism-related genes, dominated by *NIS*, is generally considered to be associated with activation of the MAPK pathway. RR-DTC is associated with downregulation of the iodine metabolism-related genes *NIS*, *TSHR*, *TPO*, and *TG* [19]. Malignant progression and reduced iodine uptake in TC are thought to be largely due to mutations in the B-Raf proto-oncogene (*BRAF*) and the telomerase reverse transcriptase promoter [28, 29]. The most frequent mutation in the *BRAF* gene is V600E (*BRAF* V600E) [30], which can cause a decrease in the iodine uptake of PTC and affect the expression of the above iodine metabolism-related genes [31, 32], as well as activating the MAPK pathway [33]. The current results showed that PC promoted the aggressiveness of PTC and significantly reduced the expression of iodine metabolism-related genes. These findings were similar to the reductions in NIS and TPO expression during iodine uptake in PTC after *BRAF* V600E mutation [32, 34].

NIS expression on the cell membrane was also significantly increased after PC knockdown, leading to enhanced iodine uptake, and indicating an inhibitory effect of PC on iodine uptake by PTC. The TPC-1 and KTC-1 cell lines selected in this study had chromosomal rearrangements of *RET*/*PTC* and the *BRAF* V600E mutation, respectively, and the effect of high PC expression on iodine uptake in PTC cells in the presence of these genetic backgrounds suggested that PC may be a common feature in the malignant transformation of PTC. We also showed that PC promoted expression of the MAPK signaling pathway. When ERK1/2 signaling was inhibited, the iodine-uptake capacity and expression levels of iodine metabolism-related genes were restored in PC-overexpressing cell lines, and the cell proliferation, invasion, and migration capacities were inhibited, indicating that PC promotes the dedifferentiation of PTC by activating the MAPK pathway. Regarding the role of PC in energy metabolism, ATP production is increased in response to elevated PC expression, and given that ATP is a frequent phosphate donor for different protein kinases [35], we speculated that PC might affect tumor progression and the expression of iodine metabolism-related genes and iodine uptake in TC cells by accelerating tumor energy metabolism to increase ATP production and activate the MAPK/ERK signaling pathway. Although PC is known to regulate energy metabolism, changes in iodine metabolism-related genes expression are considered to be likely due to secondary effects. Meanwhile, PC was previously shown to protect against oxidative stress in tumor cells [36, 37], while other studies found that PC activity was increased by increased ROS [38]. Previous studies also showed that the MAPK/ERK signaling pathway in TC cells could be blocked through elevated intracellular ROS levels, while the expression of iodine metabolism-related genes could also be affected by ROS [39]. Further studies are therefore needed to determine if PC affects the MAPK pathway via protecting against oxidative stress.

We inhibited PC activity using the small-molecule inhibitor, ZY-444, to explore the possible therapeutic value of PC for the treatment of TC. ZY-444 significantly inhibited MAPK signaling and restored the expression of *TSHR*, *NIS*, *TPO*, and *TG*, and iodine uptake, which were originally inhibited by PC, as well as reducing the aggressiveness of PTC cells. Lin et al. showed that ZY-444 could also reduce the malignant biological behaviors of breast cancer cells, such as invasion and metastasis [17]. These results further demonstrated that PC plays a role in promoting the malignant progression of tumors.

Kinase inhibitors such as sorafenib and lenvatinib inhibit tumor growth by inhibiting tumor angiogenesis. However, although these agents have demonstrated some efficacy in the treatment of advanced TC [40, 41], they are also associated with drug resistance and adverse reactions [41–43]. There is thus a need to develop more effective drugs for the treatment of advanced TC and/or to improve or restore the iodine uptake-ability of TC, to improve the success of radioactive iodine therapy and the therapeutic efficacy against dedifferentiated or RR-DTC. We revealed novel information regarding the diagnosis and treatment of PTC, which may complement the detection of *BRAF* V600E and the telomerase reverse transcriptase promoter mutations to provide a more accurate diagnosis of metastasis and loss of iodine uptake in patients with PTC in the clinical setting, and may also provide valuable information for further studies of the diagnosis and treatment of PTC by molecular imaging, but there is still a long way to go for further translational research.

In conclusion, the results of this study suggested that PC can significantly increase the proliferation, migration, and invasion of PTC cells and reduce the expression of the iodine metabolism-related genes *TSHR*, *NIS*, *TPO*, and *TG*, leading to reduced iodine uptake. These findings suggest that PC may be a significant factor affecting the decreased iodine-uptake capacity and the development of invasive behavior of PTC. PC acts mainly through activation of the MAPK/ERK signaling pathway. Accordingly, the PC inhibitor ZY-444 can inhibit metastasis and improve iodine uptake in PTC cells. These results thus provide a basis for clinical studies exploring the roles of PC in the decreased iodine-uptake capacity and development of invasive behavior in PTC.

## Materials and methods

### Cell lines and culture conditions

The human PTC cell lines TPC-1 and KTC-1 and human anaplastic TC cell line 8505c were obtained from the European Collection of Animal Cell Cultures (Salisbury, UK). The immortalized human thyroid epithelial cell line Nthy-ori 3-1 was purchased from the Chinese Academy of Science. All cell lines were cultured in RPMI-1640 (Gibco, USA) complete medium containing 10% fetal bovine serum (FBS, Gibco), 100 U/mL penicillin (Gibco), and 100 mol/mL streptomycin (Gibco) at 37 °C in a 5% CO_2_ environment.

### Lentivirus preparation, transfection, and screening

Using human PC gene primer sequences, PCshRNA-GFP-puro-lentivirus, scramble-GFP-puro-lentivirus, PC-OE-GFP-puro lentivirus, and vector-GFP-puro were purchased from Hanheng Biotechnology (Hanheng Biotechnology Co., Ltd., Shanghai, China). We previously injected various concentrations of GFP-labeled lentivirus into the cells and measured the effectiveness of the infection, and then applied the obtained optimal multiplicity of infection in subsequent lentivirus infection experiments [44]. TPC-1 and KTC-1 cells were infected with lentiviruses by plating in six-well plates to reach 60–80% confluence, followed by the addition of 1 mL RPMI-1640 complete culture medium containing 2 mg/mL polybrene (Hanheng Biotechnology Co., Ltd.). Lentiviral supernatant (50 μL) was then collected and added to the dish, followed by 1 mL of culture medium 4 h later. After 48 h, the culture medium was replaced with RPMI-1640 complete medium containing 1 mg/mL puromycin (Sigma-Aldrich, St. Louis, MO, USA) and cultured for 1 week for screening.

### CCK-8 assay

The Cell Counting Kit-8 (CCK-8, Beyotime, China) was used to assess cell proliferation. Cell lines were plated in five 96-well plates at 1000 cells/well and cell proliferation was assayed for 5 days. CCK-8 reagent (10 μL) was added to each well and the absorbance at 450 nm was measured using a plate reader (Multiskan MK3, Thermo Fisher Scientific, MA, USA).

### Clonogenic assay

Cell colony-forming capacity was detected using plate cloning assay. Seven hundred cells were seeded into six-well plates and cultured for 7 consecutive days. The cells were then collected, fixed in 4% paraformaldehyde, stained with crystal violet, and washed with PBS. Colonies comprising >50 cells were counted as a clone [45].

### Cell migration and invasion assays

Cell migration and invasion assays were carried out using transwell chambers (pore size 8 μm; Corning, USA,3422) and BioCoat Matrigel-coated inserts with BioCoat chambers (BD Bioscience, Franklin Lakes, NJ, USA,354480), respectively [46]. Cells were initially starved on day 1 by culture in FBS-free RPMI-1640 medium. On day 2, 2.5 × 10^5^ cells/well were resuspended in 200 μL (migration assay) or 500 μL (invasion assay) of serum-free medium and inoculated into the upper chamber, while the lower chamber contained 500 μL of RPMI-1640 medium containing 10% FBS (migration assay) or 750 μL of RPMI-1640 medium containing 20% FBS (invasion assay). Cells were incubated at 37 °C in a 5% CO_2_ incubator for 24 and 48 h for migration and invasion assays, respectively. Cells in the upper chamber were carefully removed and cells on the surface of the lower chamber were fixed with 4% paraformaldehyde, stained with crystal violet, and washed with Dulbecco’s PBS. Five randomly selected fields of view were examined under a light microscope (Carl Zeiss, Germany) and the migrating cells were photographed and counted. We performed Cell migration and invasion assays at 0.5, 1, and 1.5 µM concentration of ZY-444 gradients. There were significant decreases in the numbers of invasive and migrating cells at 1 µM ZY-444.

### Wound-healing assay

Cells were seeded into six-well plates to reach 95% confluence. A 200-μL pipette tip was then used to draw a vertical line on the culture dish, and the cells were washed two to three times with PBS to remove detached cells. Each well was observed and photographed under an inverted microscope at 0 and 24 h (Carl Zeiss), and the characteristics of the scratch were measured using ImageJ software. The cell-migration rate was calculated according to the following formula: [(width_0h_ − width_24h_)/width_0h_] × 100%.

### RNA extraction and qRT-PCR

Total RNA was extracted from stably transfected cell lines using the RNeasy Mini kit (Qiagen, USA) and used to synthesize cDNA by reverse transcription using Prime Script RT Master Mix (Perfect Real Time) (Takara, Japan). qRT-PCR was performed using a SYBR Green Premix Ex Taq kit (Takara). The primers used are shown in Supplemental Table S1 [23]. PCR was carried out as follows: 95 °C for 30 s, followed by 40 cycles of 95 °C for 3 s and 60 °C for 34 s. The relative gene expression level was calculated as 2^-ΔΔCT^.

### Protein extraction and western blot analysis

Cells were lysed on ice for 20 min using RIPA lysis buffer (Beyotime) with protease inhibitor cocktail (Sangon Biotech, China) and the protein lysates were collected. The protein concentration of each sample was determined using a Pierce BCA protein detection kit (Thermo Fisher Scientific). Protein lysates were boiled in 5× protein-loading buffer (Beyotime) at 95 °C for 10 min for denaturing, and then separated by 4–12% sodium dodecyl sulfate-polyacrylamide gel electrophoresis. The bands were transferred to a polyvinylidene difluoride membrane (Millipore, USA), blocked with 5% skim milk for 1 h, and the membrane was incubated with primary antibodies at 4 °C overnight. The membrane was then washed three times with Tris-buffered saline with Tween 20 (TBST), incubated with horseradish peroxidase-labeled secondary antibodies (Beyotime) at room temperature for 2 h, and washed three times with TBST. The protein bands were then visualized with ECL reaction reagents (Beyotime) using a Western Lightning Gel Imaging System (Tanon 4600; Tianneng Technology Corporation, Shanghai, China). Densitometric analysis of individual blot signals was carried out based on three independent western blot experiments using Image J software.

### Immunofluorescence assay

A slide was placed in a six-well plate and cells were inoculated on a coverslip. Each well was then fixed with 4% paraformaldehyde overnight. The cells were incubated with NIS primary antibody anti-NIS (Invitrogen, CA, USA, MA5-12308) at 4 °C overnight, the primary antibody was aspirated on the 3rd day, and the cells were washed with PBS for 30 min. After incubation with secondary antibody (Beyotime) at 37 °C for 1 h, the cells were stained with 4′,6‐diamidino‐2‐phenylindole for 15 min and washed with PBS. Finally, the slides were mounted for observation under a fluorescence microscope (Carl Zeiss) and the fluorescence intensity was analyzed using ImageJ software. The relative fluorescence intensity was calculated by dividing the fluorescence intensity of the cells by the fluorescence intensity of the background.

### Radioiodine-uptake assay

Iodide-uptake assays were performed as described previously [47]. The cells were seeded in 24-well plates with 5 × 10^4^ cells/well for 24 h, washed three times with PBS, and 500 μL of a solution containing 0.1 µCi ^125^I- NaI and 10 µM NaI were added to each well. The cells were incubated for 0, 5, 10, 15, 30, 45, 60, 90, or 120 min and then washed three times with ice-cold PBS, followed by the addition of 500 µL of sodium hydroxide solution per well to lyse the cells. After 15 min, the radioactivity counts/min were measured using a gamma radiometer (Rihuan Technology Investment Co., Ltd., Shanghai, China). Viable cells were counted using trypan blue (Beyotime), and there was no significant difference in the total number of cells between groups [48].

### siRNA and RNA interference

ERK1 and ERK2 siRNAs and non-targeting siRNAs were constructed by GenePharma (Shanghai, China) (Supplemental Table S2). The siRNAs were transfected using Lipofectamine RNAiMAX transfection reagent (Invitrogen). The cells were seeded in six-well plates and cultured overnight. The siRNAs were diluted in Opti-MEM serum-free medium followed by the addition of Lipofectamine RNAiMAX (Invitrogen). After incubation for 20 min, the transfection mixture was added to the cells and incubated for 6 h. The mRNA expression levels of ERK1 and ERK2 were detected by qRT-PCR after 24 h of culture in RPMI 1640 complete medium, and the protein expression levels were detected by western blot after 72 h of culture.

### Chemicals and reagents

ZY-444 was a gift from Professor Yihua Chen’s group at East China Normal University, Shanghai, China [17]. SCH772984 (ERK1/2 inhibitor, S7101) was purchased from Selleck, Shanghai, China. All drugs were dissolved in dimethyl sulfoxide solution.

The antibodies used in this study were: anti-PC (Santa Cruz Biotechnology, USA, sc-271493), anti-TSHR (Beyotime, AF1186), anti-NIS (Invitrogen, MA5-12308), anti-TPO (Abcam, UK, ab133322), anti-TG (Santa Cruz Biotechnology, sc-365997), anti-phospho-ERK1/2 (Cell Signaling Technology, USA, 4370), anti-ERK1/2 (Cell Signaling Technology,4695), anti-phospho-MEK1/2 (Cell Signaling Technology, 9154), anti-MEK1/2 (Cell Signaling Technology, 4694), anti-GAPDH (Beyotime, AF1186), and anti-α-tubulin (Beyotime, AF5012).

### PC activity assay

PC activity was assessed using a PC kit according to the manufacturer’s instructions (Comin Biotechnology Co., Ltd. Suzhou, China). Briefly, cells were treated with dimethyl sulfoxide or a range of concentrations of ZY-444 for 24 h. The cells (6 × 10^6^) were then washed with PBS and 1 mL extract buffer (50 mM Tris-HCl, pH 7.4 containing 2 mM magnesium acetate, 1 mM EDTA, and 10% glycerol) was added, the cells were collected and lysed using a homogenizer in an ice bath, and insoluble particles were removed by differential centrifugation (600 × *g*, 5 min, 4 °C). The supernatant was then transferred to another microtube and centrifuged at 11,000 × *g* for 10 min at 4 °C. The precipitate was suspended in 1 mL extract buffer and sonicated at 200 W output in an ice bath with 30× 3 s pulses with 10 s intervals. A working solution was prepared by mixing malate dehydrogenase, acetyl coenzyme, and NADH, and incubated at 37 °C for 5 min. The reaction solution was created by combining 10 μL sample, 10 μL reagent (100 mM triethanolamine buffer pH 8.0 containing 30 mM ATP and 450 mM sodium bicarbonate), and 180 μL working solution in a 96-well plate and the initial absorbance value A1 and the absorbance value A2 after 2 min were detected using a microplate reader. PC activity (nmol/min/10^4^ cells) was calculated as 6.43 × (A1−A2).

### Animal studies

Animal experiments were performed according to the Guide for the Care and Use of Laboratory Animals (NIH publication nos. 80-23, revised 1996) and in compliance with the Ruijin Hospital Animal Care and Use Guidelines for animal experimentation.

Establishment of tumor xenograft mice: Four-week-old female BALB/c nude mice (Vital River Laboratory Animal Technology Co., Ltd. China) were used to establish examine the behavior of tumor xenografts. Animals were maintained under specific pathogen-free conditions. All mice were randomized to each group. A total of 1 × 10^7^ cells were suspended in PBS and injected subcutaneously into the right side of the mice. Tumor volume was measured every 3 days using calipers, and the tumor volume was calculated according to the formula: (*D* × *d*^2^)/2, where *D* was the largest diameter of the tumor and *d* was the smallest diameter. The mice were killed on day 12 and the tumor size was assessed. Each group included five mice.

The in vivo metastatic abilities of the cells were examined by tail vein injection into mice [49]. About 5 × 10^5^ cells were injected via the tail vein into 4-week-old female BALB/c nude mice (*n* = 8 mice per cell line) and tumor growth was monitored for 6 weeks, or until the mice reached humane endpoints (i.e., signs of severely labored breathing, rapid weight loss, hunched posture, and moribund status). The lungs and liver were harvested, fixed in 4% paraformaldehyde, embedded in paraffin, cut into 4-μm sections, and stained with hematoxylin and eosin according to a standard protocol. The slides were observed and photographed under an optical microscope (Carl Zeiss).

Iodine uptake assay of xenograft tumors: Mice were injected with 100 μL solution containing ~100 μCi ^125^I-NaI via the tail vein for 120 min. The tumor tissue was then placed in a pre-weighed and labeled γ-counting tube and reweighed. The radioactivity of the tissue was measured and expressed as the ratio of radioactivity contained in each gram of tissue to the total radioactivity injected, i.e., %ID/g. Each group included three mice [50].

### Statistical analysis

All data are presented mean ± standard error. Analysis was carried out using GraphPad Prism 6 software. Differences between two groups were analyzed by Student’s *t*-tests and differences among three or more groups were analyzed by analysis of variance. Categorical data were compared using *χ*^2^ tests. The results are expressed as the mean ± standard deviation (SD) of three independent experiments. A *p*-value < 0.05 was considered statistically significant.

## Supplementary information

Table S1 Real-time PCR primers used in the study.

Table S2 RNA oligo sequence used in the study

Figure S1.PC promotes the aggressiveness of PTC cells.

Figure S2.PC reduces the expression of iodine metabolism genes, iodine uptake, and promotes MAPK pathway signaling in PTC cells.

Figure S3.ZY-444 inhibits the cell viability of PTC cells.

Figure S4ZY-444 restores the aggressiveness of PC-overexpressed PTC cells.

Figure S5.ZY-444 restores the expression of iodine metabolism genes and iodine uptake of PC-overexpressed PTC cells.

Figure S6.Quantitative analysis of the protein expressions of TSHR, NIS, TPO, TG, ERK1/2, pERK1/2 in PC-overexpressed TPC-1 and KTC-1 cells with siRNA or SCH772984 knockdown ERK1/2 signaling.

Supplymentary- Figure legends

Original Data File

## Data Availability

The authors declare that data supporting the findings of this study are available within the article.
