# Supplementary material for: Pyruvate carboxylase promotes malignant transformation of papillary thyroid carcinoma and reduces iodine uptake
Source: Cell Death Discov. 2022 Oct 20;8:423. doi: 10.1038/s41420-022-01214-y (PMC9585021; doi:10.1038/s41420-022-01214-y)
Supplement: Supplementary file 1 — Table S1 Real-time PCR primers used in the study. [file 41420_2022_1214_MOESM1_ESM.docx]

Table S1 Real-time PCR primers used in the study.

| Gene | Primer sequences (5′ to 3′) |
| --- | --- |
| *PC* | F: 5′-ATGTTGCCCACAACTTCAGCAAGC-3′ |
|  | R: 5′-AGTTGAGGGAGTCAAACACACGG-3′ |
| *TSHR* | F: 5′- GATATTCAACGCATCCCCAG-3′ |
|  | R: 5′-AGCTGCTGCAGAGTCACATC-3′ |
| *NIS* | F: 5′-CTGCCCCAGACCAGTACATGCC-3′ |
|  | R: 5′-TGACGGTGAAGGAGCCCTGAAG-3′ |
| *TPO* | F: 5′-ACTTGGATCTCCATGTCGCT-3′ |
|  | R: 5′-GCAGTGTGGATTTAGTGCCA-3′ |
| *TG* | F: 5′-CACCAACTCCCAACTTTTCC-3′ |
|  | R: 5′-CAACTGACCTCCTTTGCCA-3′ |
| *ERK1* | F: 5′-CCCCCTAGCCCAGACAGACAT-3′ |
|  | R: 5′-GGCTGGGCACAGTGTCCATT-3′ |
| *ERK2* | F: 5′-CTGTTCCCAAATGCTGACTCCAA-3′ |
|  | R: 5′-CTCGTCACTCGGGTCGTAA-3′ |
| *actin* | F: 5′-GCACAGAGCCTCGCCTT-3′ |
|  | R: 5′-GTTGTCGACGACGAGCG-3′ |
