# Supplementary material for: Pyruvate carboxylase promotes malignant transformation of papillary thyroid carcinoma and reduces iodine uptake
Source: Cell Death Discov. 2022 Oct 20;8:423. doi: 10.1038/s41420-022-01214-y (PMC9585021; doi:10.1038/s41420-022-01214-y)
Supplement: Supplementary file 2 — Table S2 RNA oligo sequence used in the study [file 41420_2022_1214_MOESM2_ESM.docx]

Table S2 RNA oligo sequence used in the study

| Gene | RNA oligo (5′ to 3′) |
| --- | --- |
| Negative control | Sense: 5′-UUCUCCGAACGUGUCACGUTT-3′ |
|  | Anti-Sense: 5′-ACGUGACACGUUCGGAGAATT-3′ |
| ERK1-homo225 | Sense: 5′-ACACGCAGUUGCAGUACAUTT-3′ |
|  | Anti-Sense: 5′-AUGUACUGCAACUGCGUGUTT-3′ |
| ERK1-homo888 | Sense: 5′-GACCGGAUGUUAACCUUUATT-3′ |
|  | Anti-Sense: 5′-UAAAGGUUAACAUCCGGUCTT -3′ |
| ERK2-homo513 | Sense: 5′-CACCAACCAUCGAGCAAAUTT-3′ |
|  | Anti-Sense: 5′-AUUUGCUCGAUGGUUGGUGTT-3′ |
| ERK2-homo1171 | Sense: 5′-CCAUAUCUGGAGCAGUAUUTT-3′ |
|  | Anti-Sense: 5′-AAUACUGCUCCAGAUAUGGTT-3′ |
