# Supplementary figures and images for: Pyruvate carboxylase promotes malignant transformation of papillary thyroid carcinoma and reduces iodine uptake
Source: Cell Death Discov. 2022 Oct 20;8:423. doi: 10.1038/s41420-022-01214-y (PMC9585021; doi:10.1038/s41420-022-01214-y)

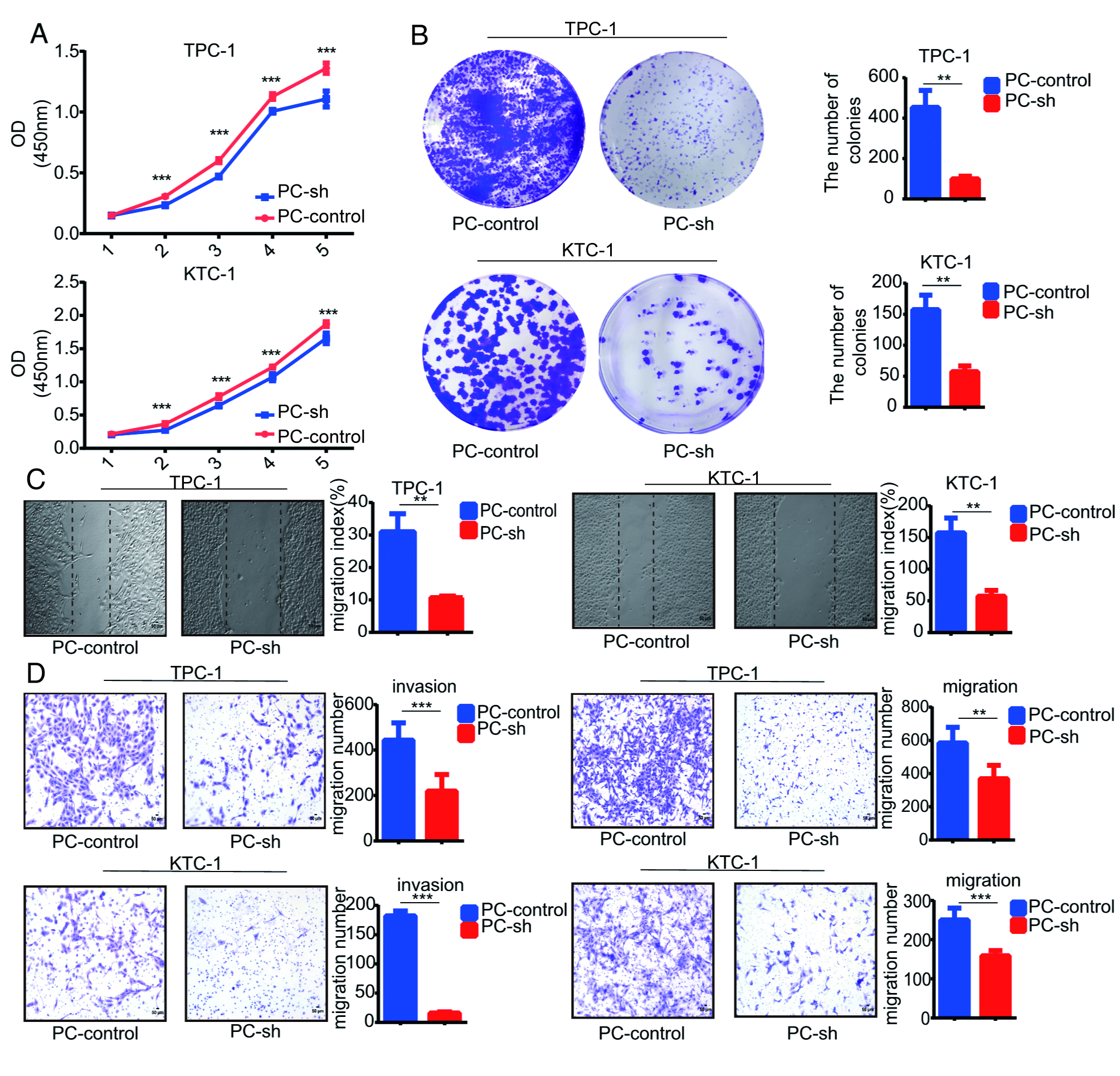

Supplement: Supplementary file 3 — Figure S1.PC promotes the aggressiveness of PTC cells. [file 41420_2022_1214_MOESM3_ESM.tif]

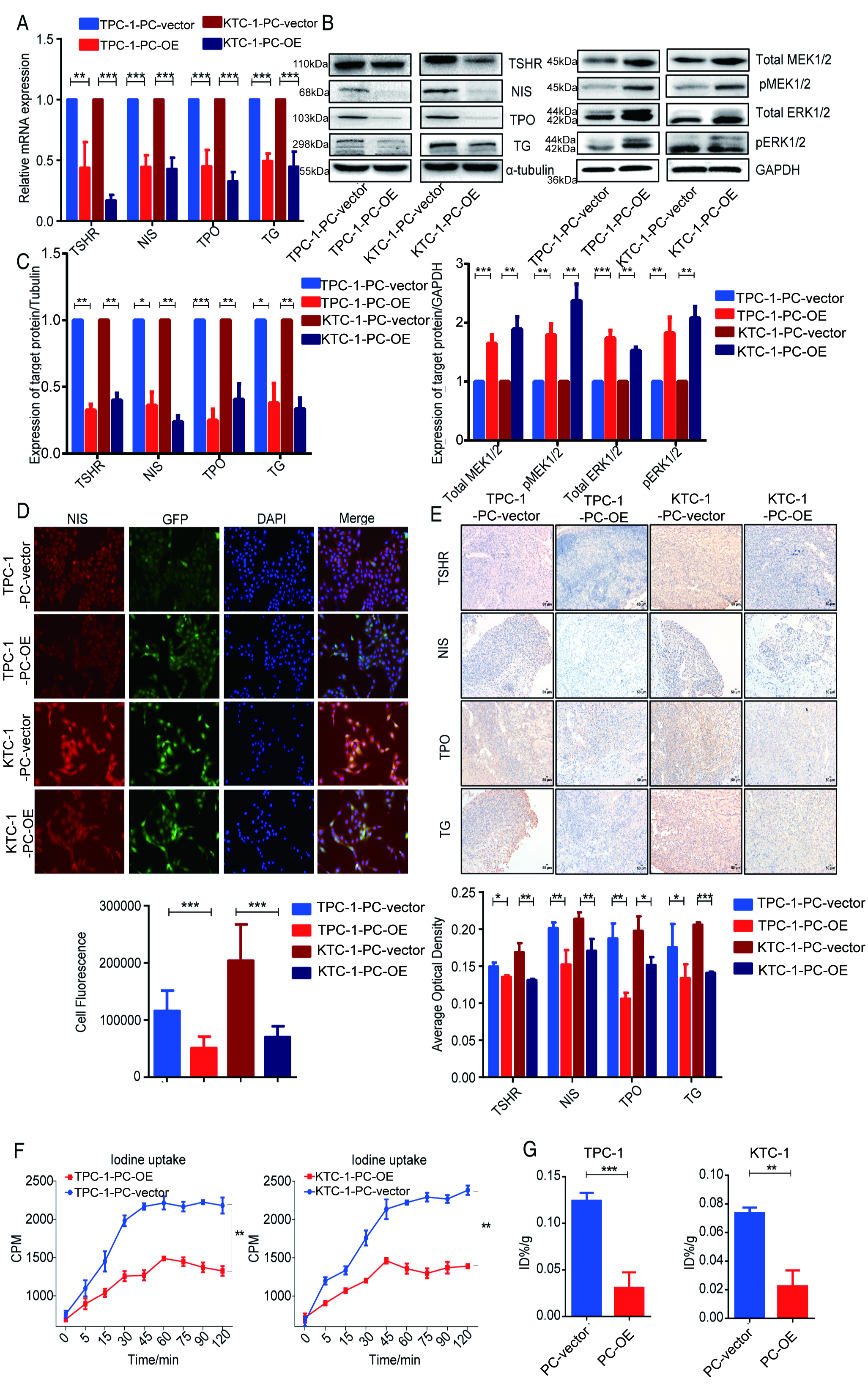

Supplement: Supplementary file 4 — Figure S2.PC reduces the expression of iodine metabolism genes, iodine uptake, and promotes MAPK pathway signaling in PTC cells. [file 41420_2022_1214_MOESM4_ESM.tif]

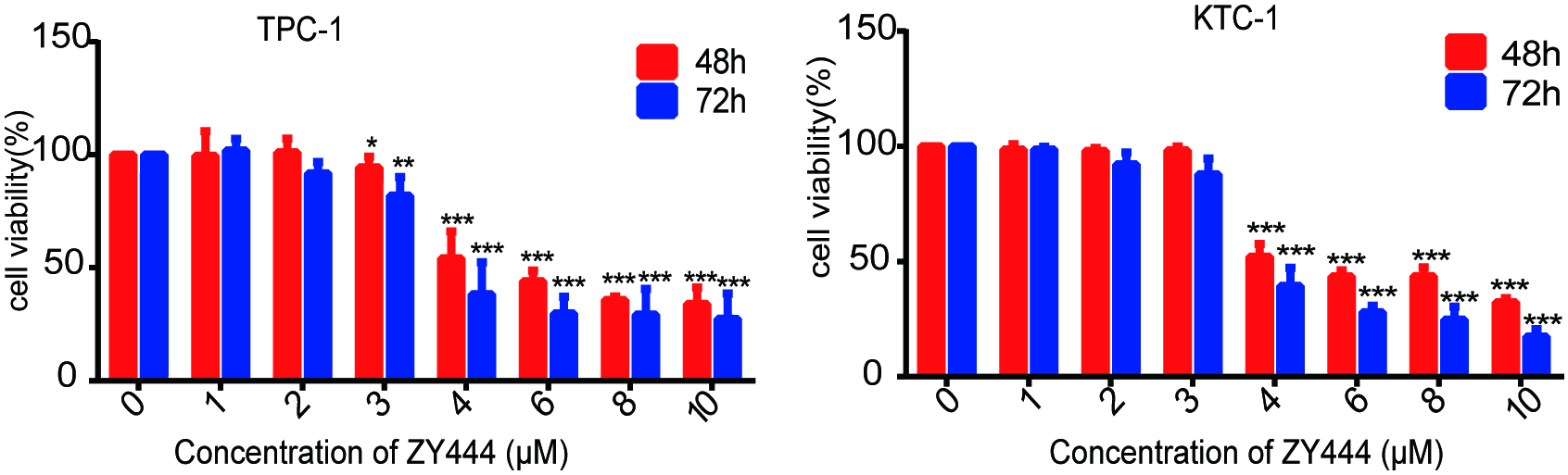

Supplement: Supplementary file 5 — Figure S3.ZY-444 inhibits the cell viability of PTC cells. [file 41420_2022_1214_MOESM5_ESM.tif]

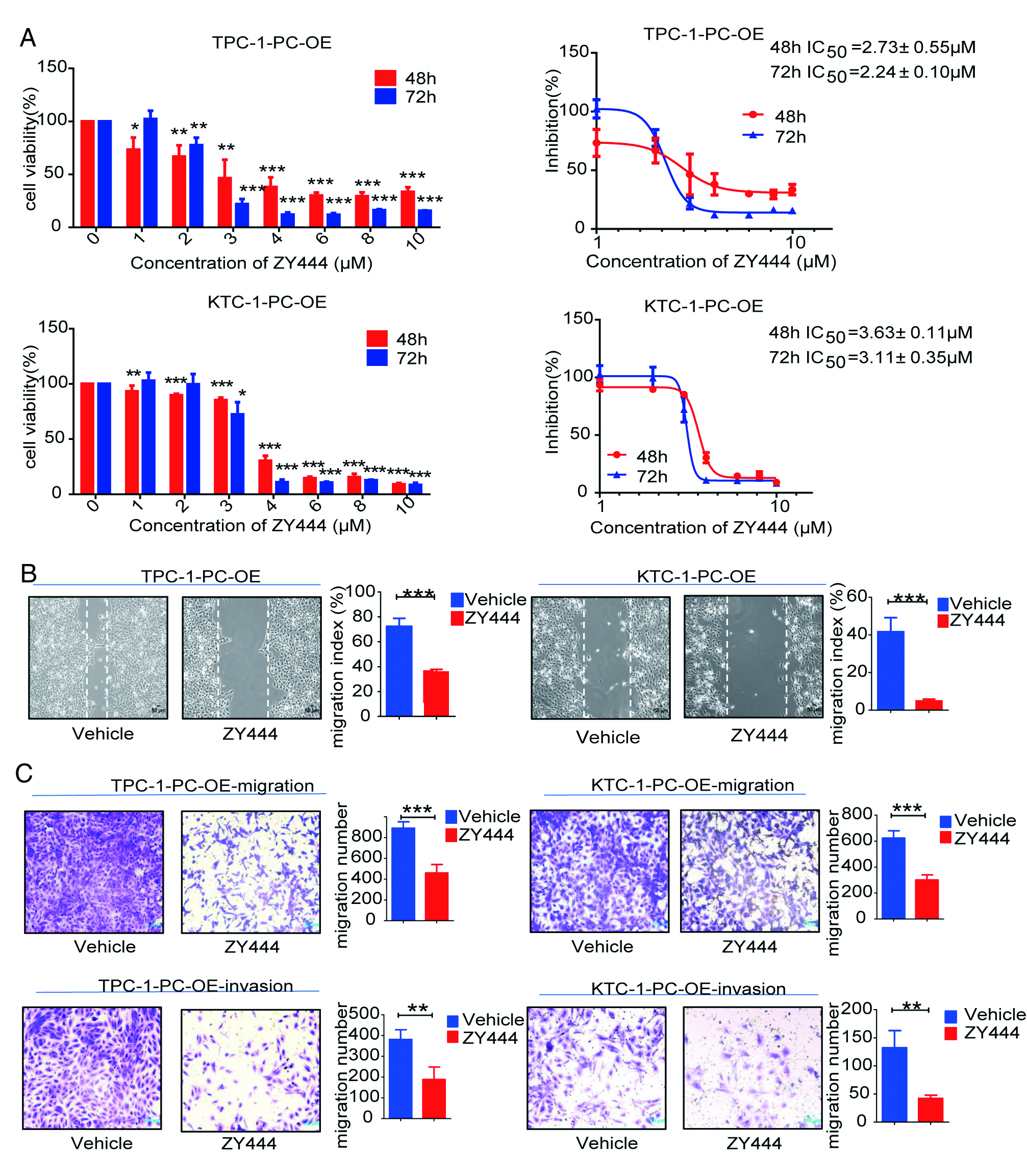

Supplement: Supplementary file 6 — Figure S4ZY-444 restores the aggressiveness of PC-overexpressed PTC cells. [file 41420_2022_1214_MOESM6_ESM.tif]

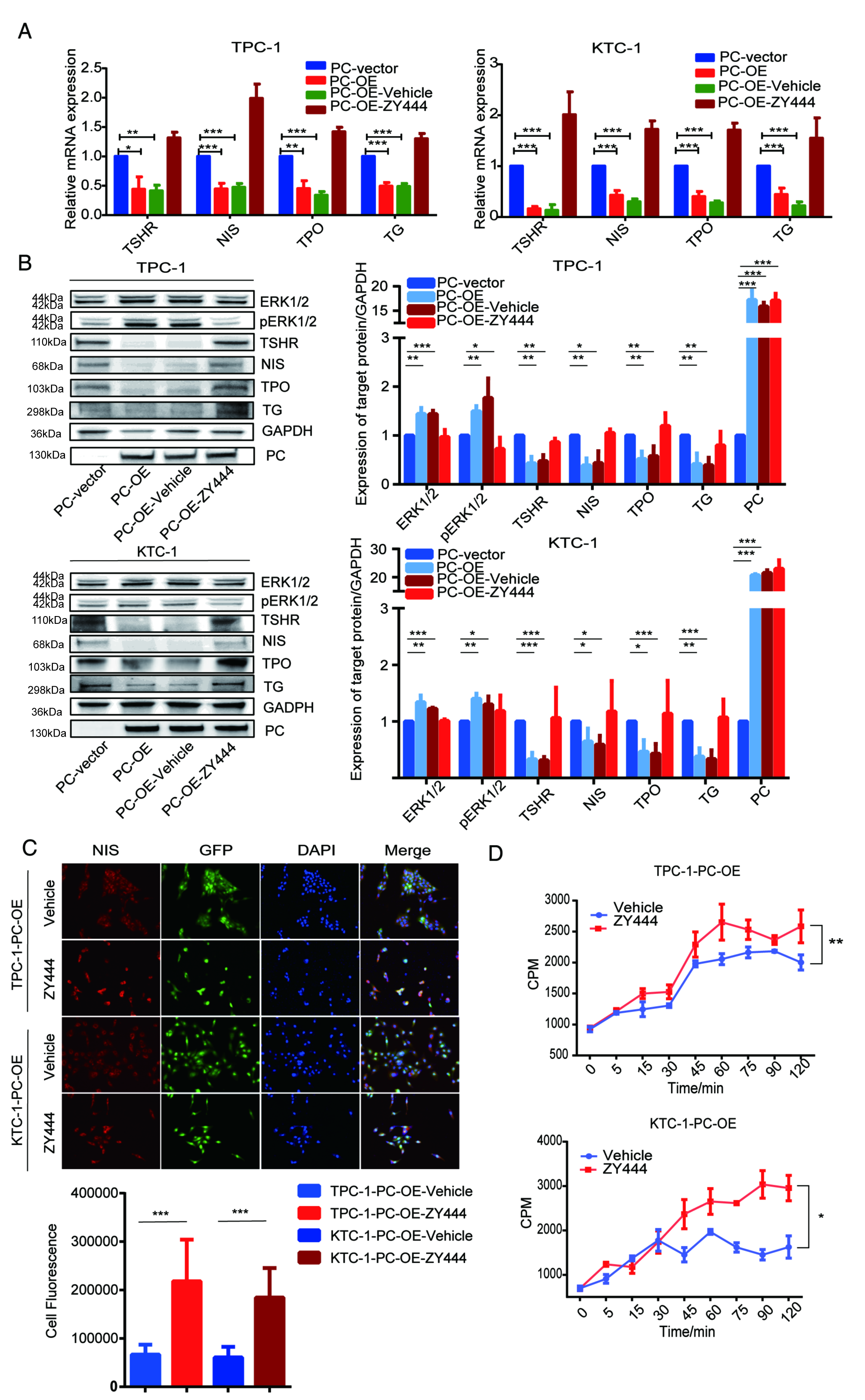

Supplement: Supplementary file 7 — Figure S5.ZY-444 restores the expression of iodine metabolism genes and iodine uptake of PC-overexpressed PTC cells. [file 41420_2022_1214_MOESM7_ESM.tif]

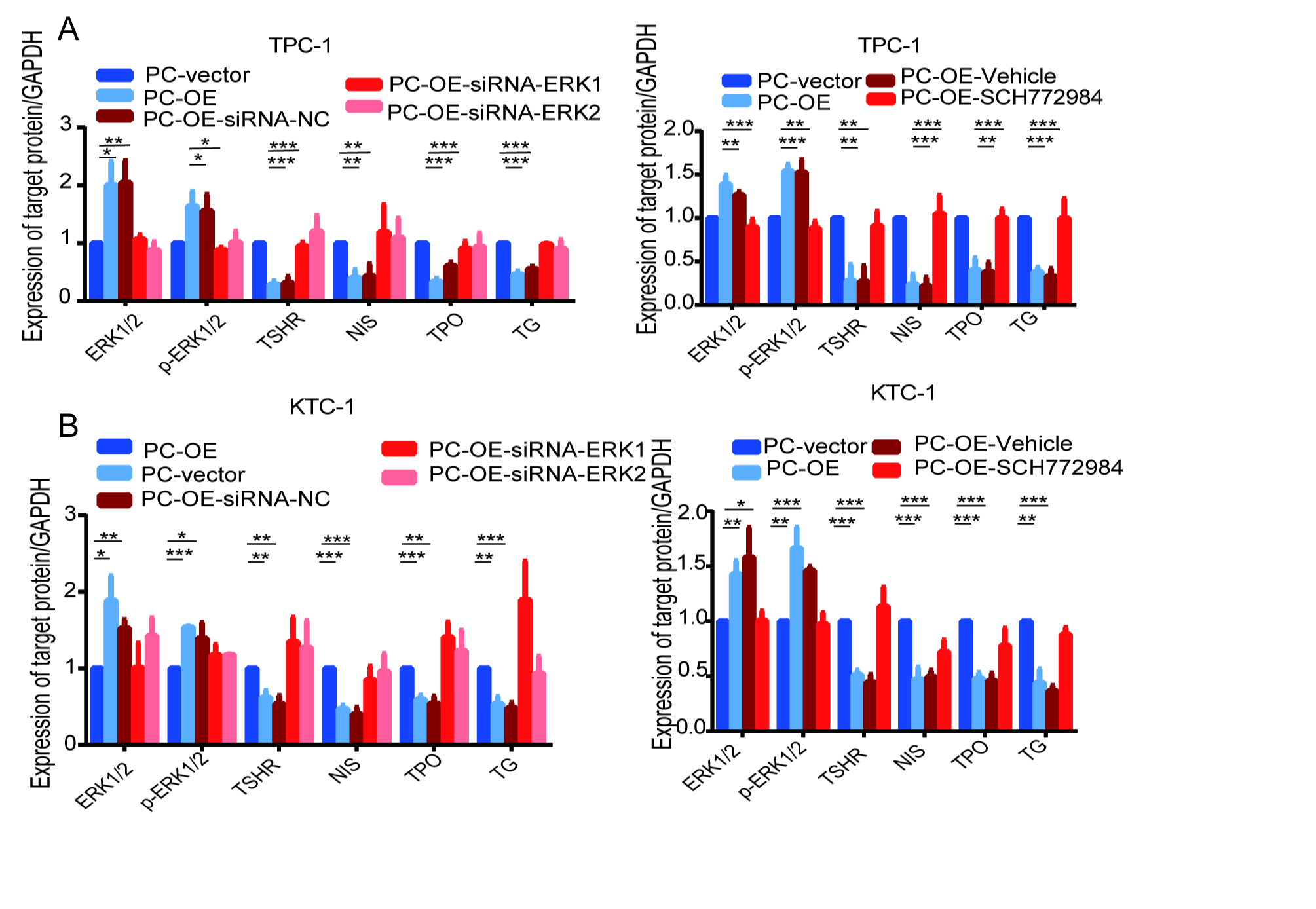

Supplement: Supplementary file 8 — Figure S6.Quantitative analysis of the protein expressions of TSHR, NIS, TPO, TG, ERK1/2, pERK1/2 in PC-overexpressed TPC-1 and KTC-1 cells with siRNA or SCH772984 knockdown ERK1/2 signaling. [file 41420_2022_1214_MOESM8_ESM.tif]
