# Supplementary material for: Pyruvate carboxylase promotes malignant transformation of papillary thyroid carcinoma and reduces iodine uptake
Source: Cell Death Discov. 2022 Oct 20;8:423. doi: 10.1038/s41420-022-01214-y (PMC9585021; doi:10.1038/s41420-022-01214-y)
Supplement: Supplementary file 9 — Supplymentary- Figure legends [file 41420_2022_1214_MOESM9_ESM.docx]

**Title: Pyruvate carboxylase promotes malignant transformation of papillary thyroid carcinoma and reduces iodine uptake**

Yang Liu,^1^ Chang Liu,^1^ Yu Pan,^1^ Jinxin Zhou,^1^ Huijun Ju,^1^ Yifan Zhang^1^*

^1^Department of Nuclear Medicine, Ruijin Hospital, Shanghai Jiaotong University, School of Medicine, No. 197, Ruijin Er Road, Shanghai 200025, China;

**Corresponding information**

Yifan Zhang, Ph.D. M.D.

Department of Nuclear Medicine

Rui Jin Hospital, Shanghai Jiao Tong University School of Medicine Shanghai, China, 200025

Tel: (+86-021) 64314813

Fax: (+86-021)64333548

Email: [zyf11300@rjh.com.cn](mailto:zyf11300@rjh.com.cn)

ORICD: 0000-0001-6488-6232

**Conflict of Interest:** The authors have no conflicts of interest to declare that are relevant to the content of this article.

**Figure S1. *PC promotes the aggressiveness of PTC cells.*** **(A),** CCK8 assay to detect the cell proliferation ability of TPC-1 and KTC-1 cells. **(B),** The clone formation capacity of TPC-1 and KTC-1 cells assessed by the clonogenic assay. **(C),** Scratch assay and **(D),** Transwell-migration and invasion assay to detect the cell metastatic ability of TPC-1 and KTC-1 cells. ***P<0.001, **P<0.01, *P<0.05, Representative micrographs of each condition taken at 100x; The results expressed as means ± standard deviation (SD) of three independent experiments and shown in histograms on the right.

**Figure S2.** ***PC reduces the expression of iodine metabolism genes, iodine uptake, and promotes MAPK pathway signaling in PTC cells.*** (**A**), RT-PCR and (**B**), Western blot to detect the mRNA and protein expressions of TSHR, NIS, TPO, TG, and MAPK pathway signals in TPC-1 and KTC-1 cells. (**C**), Quantitative analysis of protein expression measured by western blot by image J. (**D**), Immunofluorescence detection of the localization and expression of NIS in TPC-1 and KTC-1 cells by microscopy with a 20× objective lens. Quantification of NIS fluorescence shown below. (**E**), Immunohistochemistry detection of TSHR, NIS, TPO, TG expression in TPC-1 and KTC-1 cells xenograft tumors. Quantification of immunohistochemical staining shown below. (**F**), Iodine uptake by ^125^I uptake assay in TPC-1 and KTC-1 cells and (**G**), TPC-1 and KTC-1 xenograft tumors. ***P<0.001, **P<0.01, *P<0.05. Results represent three independent experiments.

**Figure S3.** ***ZY-444 inhibits the cell viability of PTC cells.*** CCK8 assay to detect the cell proliferation ability in TPC-1 and KTC-1 cells with ZY-444 treatment.

**Figure S4. *ZY-444 restores the aggressiveness of PC-overexpressed PTC cells.***

(**A**), CCK8 assay to detect the cell proliferation ability in PC-overexpressed TPC-1 and KTC-1 cells with ZY-444 treatment. The half-maximal inhibitory concentration (IC50) calculated by curve fitting. (**B**), Scratch assay and (**C**), Transwell-migration and invasion assay to detect the cell metastatic ability in PC-overexpressed TPC-1 and KTC-1 cells with ZY-444 treatment. ***P<0.001, **P<0.01, *P<0.05. Representative micrographs of each condition taken at 100x; The result expressed as means ± standard deviation (SD) of three independent experiments and shown in histograms on the right. The experimental concentration of ZY-444 used was 1uM.

**Figure S5. *ZY-444 restores the expression of iodine metabolism genes and iodine uptake of PC-overexpressed PTC cells.***

(**A**), RT-PCR to detect mRNA expressions of TSHR, NIS, TPO, TG in PC-overexpressed TPC-1 and KTC-1 cells with ZY444 treatment. (**B**), Western blot to detect protein expressions of TSHR, NIS, TPO, TG, ERK1/2, p-ERK1/2 in PC-overexpressed TPC-1 and KTC-1 cells with ZY444 treatment. (**C**), Immunofluorescence to detect the localization and expression of NIS in PC-overexpressed TPC-1 and KTC-1 cells with ZY444 treatment with a 20× objective lens. Quantification of NIS fluorescence shown below. (**D**), Iodine uptake by ^125^I uptake assay to detect iodine uptake in PC-overexpressed TPC-1 and KTC-1 cells with ZY444 treatment. ***P<0.001, **P<0.01, *P<0.05. The result expressed as means ± standard deviation (SD) of three independent experiments and shown in histograms on the right.

**Figure S6. Q*uantitative analysis of the protein expressions of TSHR, NIS, TPO, TG, ERK1/2, pERK1/2 in PC-overexpressed TPC-1 and KTC-1 cells with siRNA or SCH772984 knockdown ERK1/2 signaling.*** (**A**), The quantitative analysis of protein expression in TPC-1. (**B**), The quantitative analysis of protein expression in KTC-1. The quantitative analysis of protein expression measured by western blot through image J. The experimental concentration of SCH772984 used was 5uM. Results represent three independent experiments.
