# Supplementary material for: Pyruvate carboxylase promotes malignant transformation of papillary thyroid carcinoma and reduces iodine uptake
Source: Cell Death Discov. 2022 Oct 20;8:423. doi: 10.1038/s41420-022-01214-y (PMC9585021; doi:10.1038/s41420-022-01214-y)
Supplement: Supplementary file 10 — Original Data File [file 41420_2022_1214_MOESM10_ESM.docx]

**Title: Pyruvate carboxylase promotes malignant transformation of papillary thyroid carcinoma and reduces iodine uptake**

Yang Liu,^1^ Chang Liu,^1^ Yu Pan,^1^ Jinxin Zhou,^1^ Huijun Ju,^1^ Yifan Zhang^1^*

^1^Department of Nuclear Medicine, Ruijin Hospital, Shanghai Jiaotong University, School of Medicine, No. 197, Ruijin Er Road, Shanghai 200025, China;

**Corresponding information**

Yifan Zhang, Ph.D. M.D.

Department of Nuclear Medicine

Rui Jin Hospital, Shanghai Jiao Tong University School of Medicine Shanghai, China, 200025

Tel: (+86-021) 64314813

Fax: (+86-021)64333548

Email: [zyf11300@rjh.com.cn](mailto:zyf11300@rjh.com.cn)

ORICD: 0000-0001-6488-6232

**Conflict of Interest:** The authors have no conflicts of interest to declare that are relevant to the content of this article.


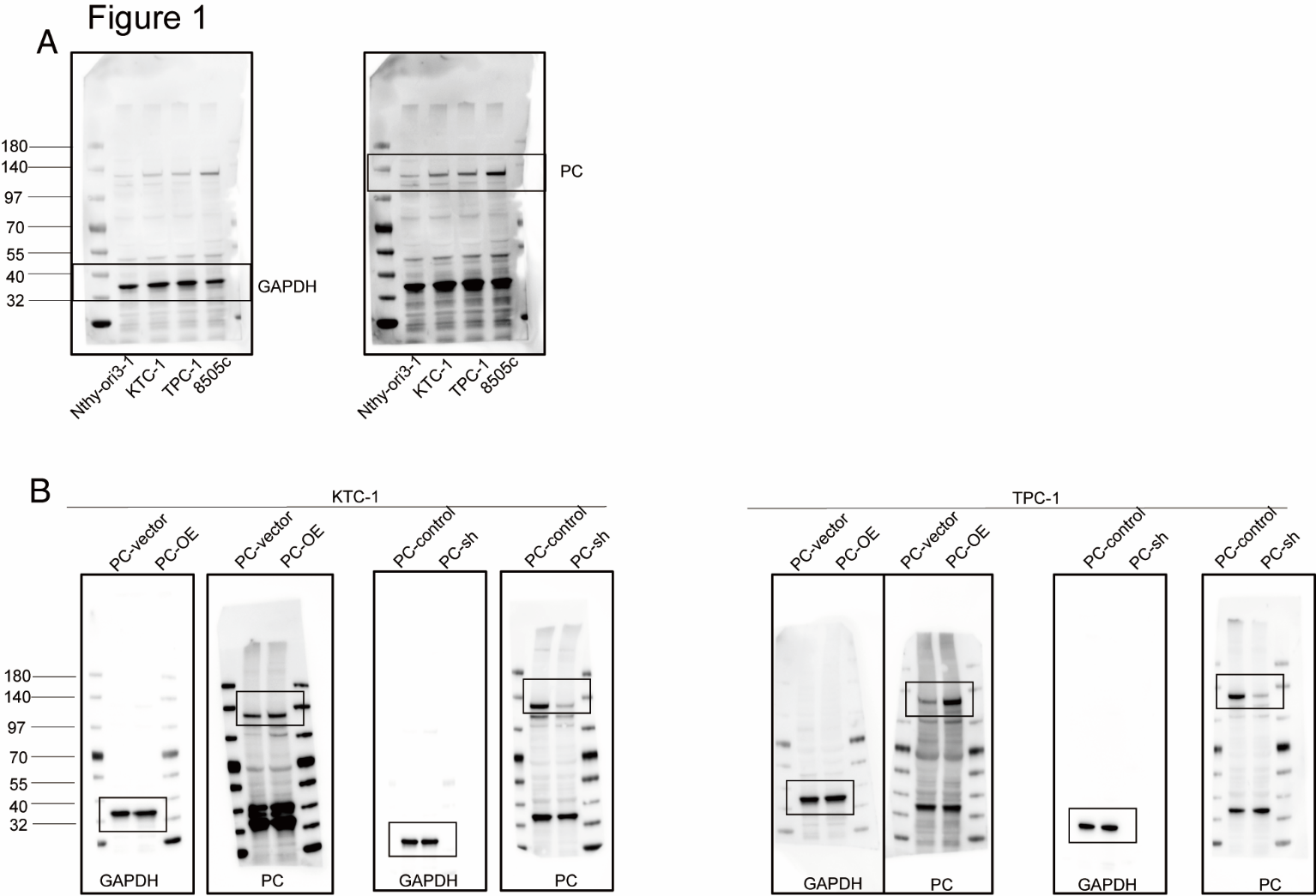


Supplementary Figure 1A&1C. The original, full blot for Figure 1A&1C.

A: PC and GAPDH expression in thyroid cancer cells, B: PC and GAPDH expression in TPC-1 and KTC-1 cells.


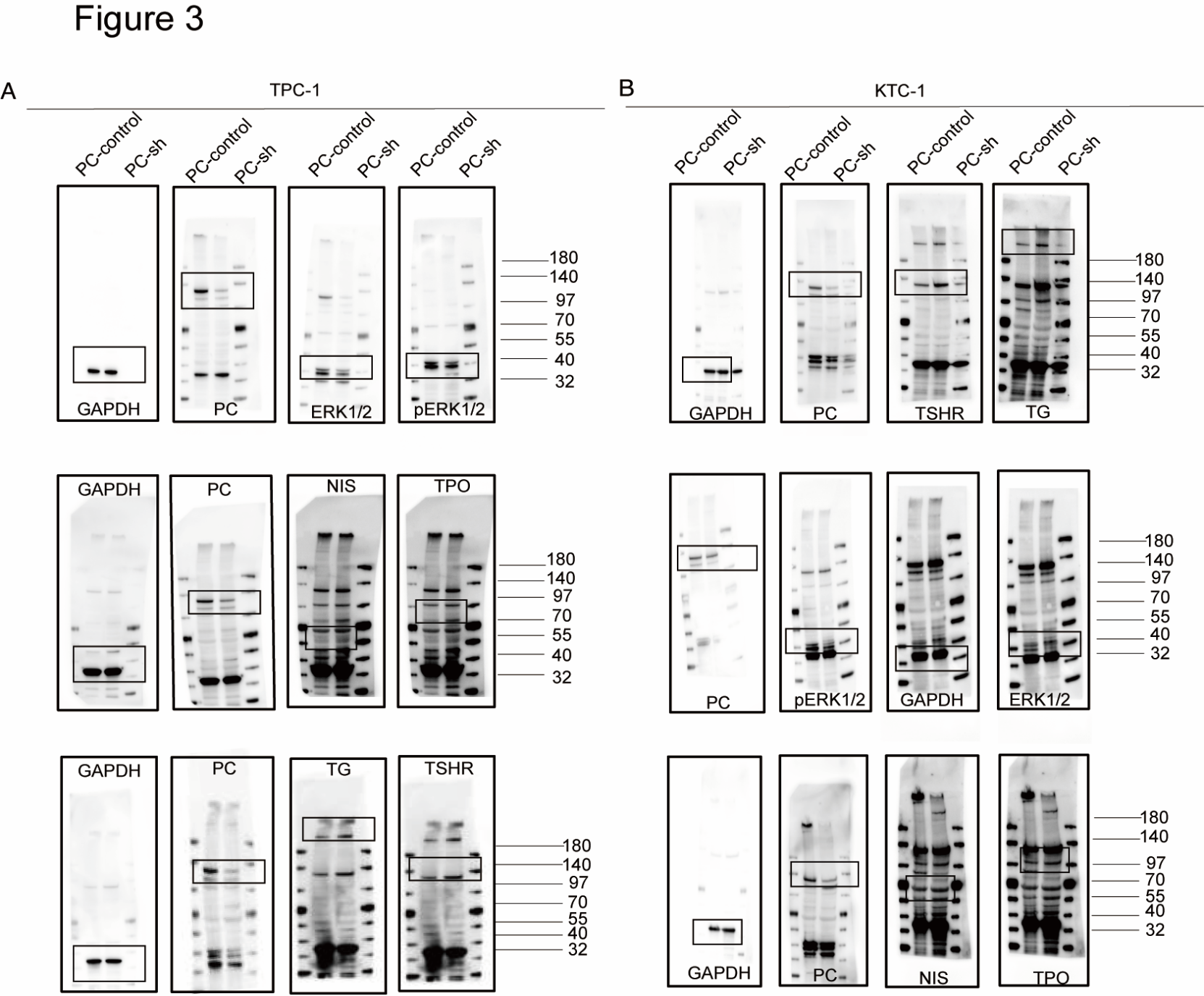


Supplementary Figure 3A. The original, full blot for Figure 3A.

A: ERK1/2, pERK1/2, TSHR, NIS, TPO, TG, and GAPDH expression in TPC-1 cells, B: ERK1/2, pERK1/2, TSHR, NIS, TPO, TG, and GAPDH expression in KTC-1 cells.


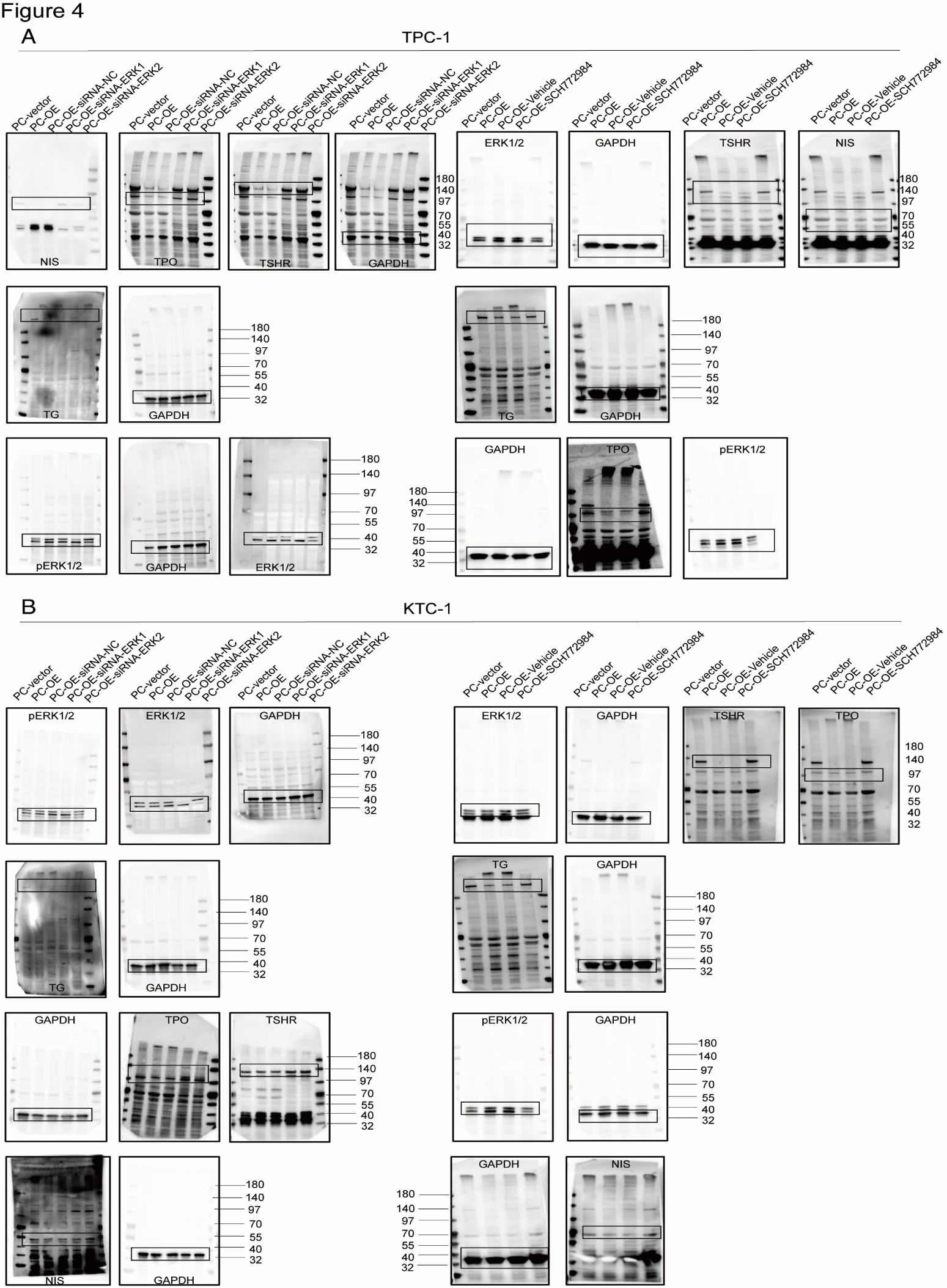


Supplementary Figure 4B. The original, full blot for Figure 4B

A: ERK1/2, pERK1/2, TSHR, NIS, TPO, TG, and GAPDH expression in TPC-1 cells, B: ERK1/2, pERK1/2, TSHR, NIS, TPO, TG, and GAPDH expression in KTC-1 cells.


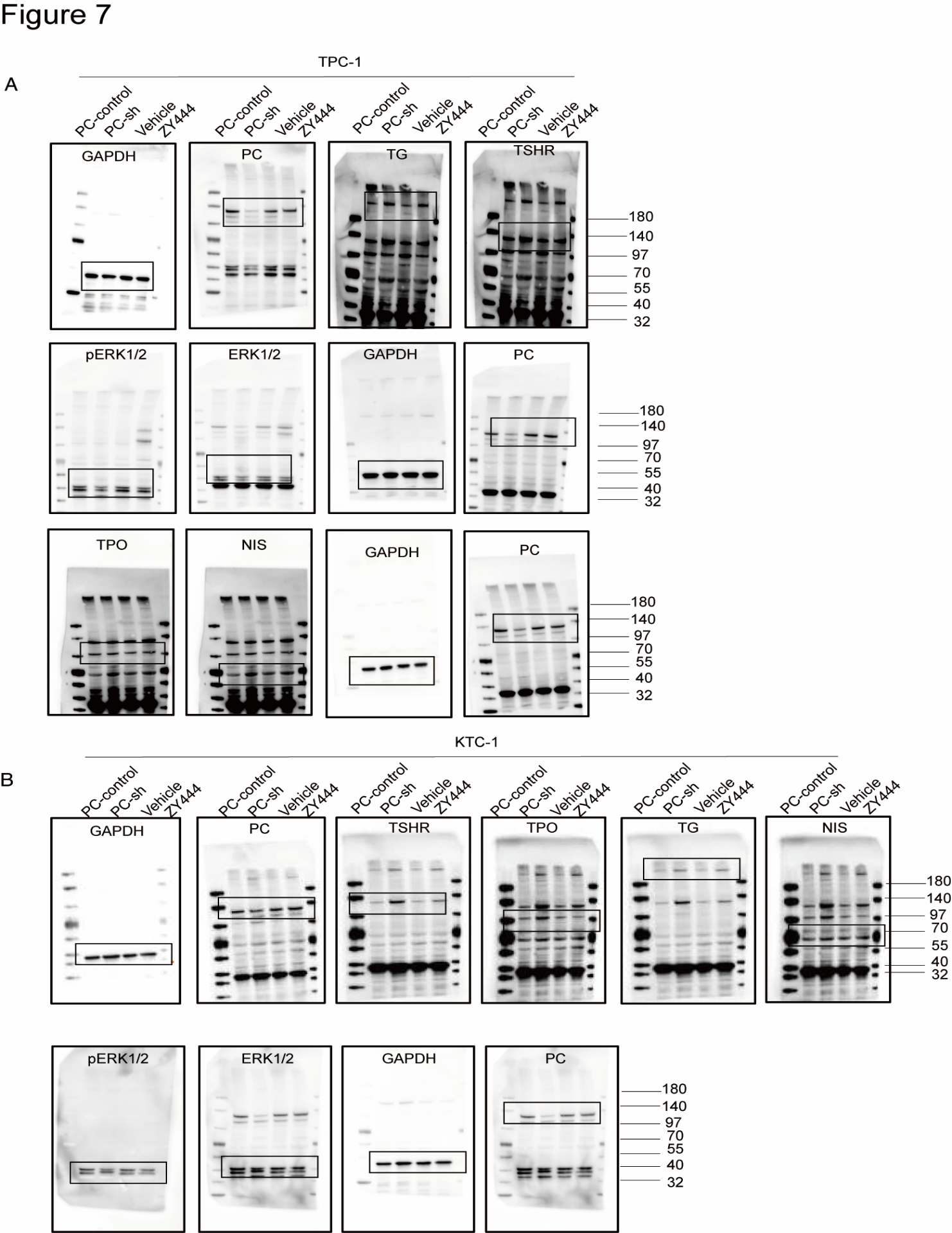


Supplementary Figure 7B. The original, full blot for Figure 7B.

A: ERK1/2, pERK1/2, TSHR, NIS, TPO, TG, and GAPDH expression in TPC-1 cells, B: ERK1/2, pERK1/2, TSHR, NIS, TPO, TG, and GAPDH expression in KTC-1 cells.
